# Multi-omics insights into co-fermentation by *Saccharomycopsis fibuligera* and *Bacillus velezensis* enhancing the nutritional, metabolic, and aromatic quality of *Pueraria thomsonii*

**DOI:** 10.3389/fnut.2026.1938235

**Published:** 2026-08-27

**Authors:** Lei Li, Xu Yang, Yibo Ning, Nana Tang, Wei Chen, Yuhan Yang, Yiduo Xue, Xiaodong Pei, Xiaoxiao Jiang, Feng Zhang, Zhifei Chen, Tao Wei

**Affiliations:** 1 China Tobacco Henan Industrial Co. Ltd., Zhengzhou, China; 2 College of Food and Bioengineering, Zhengzhou University of Light Industry, Zhengzhou, China

**Keywords:** *Bacillus velezensis*, multi-omics, *Pueraria thomsonii* Benth, *Saccharomycopsis fibuligera*, solid-state fermentation

## Abstract

**Background:**

*Pueraria thomsonii* is rich in isoflavonoids; however, its glycoside-dominated forms exhibit limited intestinal absorption and metabolism, and the material possesses undesirable sensory traits. This study employs a defined co-culture of *Saccharomycopsis fibuligera* and *Bacillus velezensis* to achieve coordinated starch hydrolysis, cell-wall degradation, and *β*-glucosidase-mediated deglycosylation. We investigate the resulting nutritional, metabolic, and volatile profiles through integrated multi-omics, establishing this consortium as a bioprocessing method for value-added *P. thomsonii*.

**Methods:**

Solid-state fermentation (SSF) was conducted at 30 °C for 72 h under microaerophilic conditions across five treatments: raw *P. thomsonii* (Y), natural fermentation (K), single-strain fermentation with *S. fibuligera* YPD01 (S) or *B. velezensis* NA03 (B), and co-fermentation with both strains at a 1:1 ratio (M). Nutritional components, total flavonoids, and total phenolics were quantified. Activities of *α*-amylase, *β*-glucosidase, and cellulase were assayed. The microbial community structure and functional genes were characterized through metagenomic sequencing. Untargeted metabolomics was performed using UPLC–MS, and volatile compounds were analyzed by GC–MS.

**Results:**

Co-fermentation achieved the highest nutritional quality, yielding reducing sugars (15.45 ± 0.26 mg/g), total flavonoids (9.30 ± 0.17 mg RUT/g), total phenolics (13.19 ± 0.25 mg GAE/g), total amino acids (52.11 ± 0.53 g/kg), and crude protein (12.56 ± 0.26%), all significantly surpassing other treatments. Both inoculated strains effectively colonized the substrate. Co-fermentation exhibited the highest activities of *β*-glucosidase (90.67 ± 2.66 U/g) and cellulase (343.77 ± 10.75 U/g). Metagenomic analysis generated approximately 659 million reads, identifying 7,737 KEGG entries, with enriched CAZy families in co-fermentation. Untargeted metabolomics identified 1,693 metabolites, with co-fermentation uniquely enriching isoflavone aglycones, peptides, and esterase-related compounds. GC–MS analysis revealed that co-fermentation produced the highest levels of fruity esters, including ethyl linoleate (1009.73 ± 32.51 μg/g) and ethyl palmitate (335.85 ± 9.76 μg/g), while hexanal was eliminated in all fermented groups.

**Conclusion:**

The *S. fibuligera*–*B. velezensis* consortium enhanced the nutritional, metabolic, and aromatic quality of *P. thomsonii* through enzymatic biotransformation and metabolic complementarity. Co-fermentation outperformed both natural and single-strain fermentations in the release of phenolic compounds and isoflavone aglycones, amino acid enrichment, and flavor development. These findings provide a theoretical basis and technical guidance for developing high-value fermented foods and offer a reference framework for the precision microbial transformation of medicinal and edible homologous materials.

## 1 Introduction

The root of *Pueraria thomsonii* Benth exemplifies an herb with a rich traditional history and significant modern pharmacological potential (1). It is rich in isoflavonoids, notably puerarin, daidzin, genistin, and their aglycones (2, 3), which contribute to its antioxidant, anti-inflammatory, hepatoprotective, and cardiometabolic effects (4, 5). However, the glycoside nature of these isoflavones results in poor lipid solubility and limited transmembrane absorption, restricting their physiological potential (6). Additionally, *P. thomsonii* often has a beany odor and an astringent taste, limiting its use in premium functional foods and pharmaceuticals (7). As a result, developing efficient, eco-friendly processing methods to transform components and enhance quality has become a major research focus.

Microbial fermentation technology, particularly solid-state fermentation (SSF), offers a promising method to enhance the functionality and flavor of substances used in both medicinal and food applications (8). SSF involves microbial growth and metabolic activity on moist solid substrates, facilitating the bioconversion of complex matrices. This process enriches small-molecule compounds, improves sensory attributes, and boosts nutritional value (9). During fermentation, enzymes such as *β*-glucosidase, cellulase, and amylase, secreted by microorganisms, break down macromolecules and convert glycoside-type isoflavones into more bioavailable aglycone forms (10, 11). Additionally, fermentation reduces antinutritional factors and produces novel flavor compounds, enhancing functional benefits and flavor profiles (12). The selection and combination of fermentation strains are crucial. Traditional methods often rely on natural fermentation driven by indigenous microflora or single-strain starters (13). While these methods have certain advantages, they also present significant limitations. Natural fermentation is unpredictable, prone to contamination, and results in inconsistent product quality, complicating standardization and scale-up (14). Single-strain fermentation offers more control but often lacks the complementary enzymatic activities needed for thorough substrate degradation and multifaceted biotransformation (15). This highlights a critical technological gap in developing robust, reproducible, and efficient fermentation processes for herbal substrates. Therefore, constructing defined microbial consortia composed of functionally characterized strains holds significant research value and practical potential.

A promising area in fermentation research involves using defined microbial consortia, where two or more well-characterized strains are co-cultured to promote intentional ecological interactions (16, 17). This approach surpasses the limitations of single-strain fermentation by enabling division of labor, cross-feeding, and enzyme production, steering the fermentation process toward specific goals (18). Among the microbial candidates studied, *Saccharomycopsis fibuligera* and *Bacillus velezensis* stand out for their unique application potential. *S. fibuligera* is known for robust secretion of *α*-amylase and saccharifying enzymes, efficiently converting starch into fermentable sugars like glucose and maltose (19). This process not only supports its own growth but also generates a sustained supply of fermentable sugars, aiding the growth and metabolic activity of other microorganisms. In contrast, *B. velezensis*, a well-known probiotic species, produces proteases, cellulases, and hemicellulases that break down cell wall structures, releasing embedded components (20). Notably, some *Bacillus* strains exhibit *β*-glucosidase activity, indicating a possible role in converting *P. thomsonii* isoflavones into aglycones (11).

While previous studies have examined single-strain fermentation of *P. thomsonii* using phenotypic and single-omics approaches, no study has systematically investigated the combined effects of a defined yeast–bacterium consortium on this substrate through integrated multi-omics analysis. The present study addresses this gap by employing a defined co-culture of *S. fibuligera* and *B. velezensis* and characterizing the resulting transformations through the combined lens of metagenomics, untargeted metabolomics, and volatilomics. This systems-level approach enables a mechanistic understanding of how microbial community structure, functional gene profiles, and metabolic outputs jointly determine the nutritional, functional, and aromatic quality of fermented *P. thomsonii*. This study provides theoretical support and technical guidance for developing high-activity, flavor-enhanced fermented foods from *P. thomsonii*. It also serves as a reference framework for the precision microbial transformation of other substances with medicinal and edible homologous materials.

## 2 Materials and methods

### 2.1 Materials

Mature roots of *Pueraria thomsonii* Benth. (approximately 2–3 years old, harvested post-flowering during the traditional harvest season in late autumn) were obtained from Xinyang City, Henan Province, China. They were dried at 60 °C, ground mechanically, and sifted through a 100-mesh sieve. The processed samples were stored in sealed containers at −20 °C until analysis.

### 2.2 Microorganisms

*Saccharomycopsis fibuligera* YPD01 and *Bacillus velezensis* NA03 are microbial strains preserved in our laboratory. Species identity was confirmed through molecular taxonomy: *B. velezensis NA03* was identified by 16S rRNA gene sequencing, while *S. fibuligera* YPD01 was identified by internal transcribed spacer (ITS) region sequencing. Detailed cell morphology and genetic sequencing data for these strains can be found in the Supplementary Figures S1, S2, respectively. Previous absolute quantitative analyses using real-time quantitative fluorescent PCR (RT-qPCR) on both co-culture and pure-culture samples showed a synergistic growth-promoting effect when *S. fibuligera* YPD01 and *B. velezensis* NA03 were combined (Supplementary Table S1).

### 2.3 Solid-state fermentation

Based on prior optimization experiments, we conducted SSF at 30 °C in 500-mL glass jars under microaerophilic conditions. Raw *P. thomsonii* served as the unfermented control (Y), while naturally fermented *P. thomsonii*, utilizing indigenous microflora, acted as the fermentation control (K, natural fermentation). The experimental treatments included: single-strain fermentation with *B. velezensis* NA03 (2.5 × 10^7^ CFU/g; B, bacterial fermentation), single-strain fermentation with *S. fibuligera* YPD01 (2.5 × 10^7^ CFU/g; S, yeast fermentation), and yeast-bacterial co-fermentation with both strains at a 1:1 ratio (each 1.25 × 10^7^ CFU/g; M, co-fermentation). The total microbial load was equivalent across all inoculated treatments (2.5 × 10^7^ CFU/g per treatment), ensuring that differential initial biomass did not confound comparisons of community dynamics or metabolic output. Each treatment used 200 g of dry substrate, with moisture content adjusted to 50%. To support microbial growth, 3.50% (w/w) ammonium sulfate was added as a nitrogen source. The initial pH was not adjusted, allowing fermentation to proceed at the substrate’s natural pH. After 72 h, samples were collected and stored at −20 °C for further analysis.

### 2.4 Physical and chemical properties after fermentation

#### 2.4.1 Analysis of fermented sample properties

Analyses of crude protein, crude fiber, crude fat, ash, total phosphorus, amino acids, and reducing sugars followed the Chinese National Standards: GB/T 6432–2018, GB/T 6434–2022 (5), GB/T 6433–2025 (4), GB/T 6438–2025, GB/T 6437–2018, GB/T 18246–2019 (3), and GB/T 5009.7–2016, respectively. Each fermented sample was mixed with water in a 1:10 (w/v) ratio, stirred for 30 min at room temperature, and centrifuged at 3000 × g for 10 min. The resulting supernatant was used to measure pH.

#### 2.4.2 Determination of total flavonoids and phenolic contents

Dried *P. thomsonii* (200 mg) was subjected to ultrasonic extraction at 300 W and 40 °C for 20 min using 8 mL of 95% methanol. The mixture was then centrifuged at 5000 × g for 10 min. The supernatant was evaporated to dryness using a rotary evaporator under reduced pressure at 40 °C, reconstituted with 80% methanol, and centrifuged again at 10,000 × g for 20 min. Total flavonoid content (TFC) was determined using the aluminum chloride colorimetric assay (21). In this assay, 1 mL of the supernatant reacted with sodium nitrite, aluminum chloride, and NaOH, and its absorbance was measured at 510 nm. Rutin served as the standard (y = 11.796x + 0.051, *R*^2^ = 0.9982, where y = absorbance at 510 nm, x = rutin concentration in mg/mL) (Supplementary Figure S3a), and TFC was expressed as mg rutin equivalents (RUT)/g. Total phenolic content (TPC) was measured using the Folin–Ciocalteu method (22). Here, 1 mL of the supernatant was mixed with Folin–Ciocalteu reagent and sodium carbonate, and absorbance was measured at 765 nm. Gallic acid served as the standard (y = 0.1091x + 0.0262, *R*^2^ = 0.9967, where y = absorbance at 765 nm, x = gallic acid in mg/mL) (Supplementary Figure S3b), and TPC was expressed as mg gallic acid equivalents (GAE)/g.

### 2.5 Enzyme extraction and activity assessment

Extracellular enzymes were extracted from fermented *P. thomsonii* by homogenizing the sample with distilled water in a 1:10 (w/v) ratio and shaking it at 30 °C for 4 h. The mixture was then filtered through Whatman No. 1 paper and centrifuged at 5000 × g for 10 min at 4 °C. The resulting supernatant was used for enzyme activity assays.

*α*-Amylase activity was measured spectrophotometrically using the 3,5-dinitrosalicylic acid (DNS) method, which quantifies maltose released at 575 nm (23). The maltose standard curve was y = 0.1773x + 0.0312 (R^2^ = 0.9956, where y = absorbance at 575 nm, x = maltose in mg/mL) (Supplementary Figure S3c). One unit (U) of activity was defined as the enzyme amount producing 1 μmol of maltose per minute.

*β*-Glucosidase activity was assessed using 4-nitrophenyl-β-D-glucopyranoside (PNPG) as the substrate, with p-nitrophenol release monitored at 420 nm (24). Its concentration was determined using a standard curve (y = 1.0457x - 0.0074, *R*^2^ = 0.9995, where y = absorbance at 420 nm, x = p-nitrophenol in μmol/L) (Supplementary Figure S3d). One unit (U) was defined as the enzyme amount liberating 1 μmol of p-nitrophenol per minute.

Cellulase activity was determined by hydrolyzing carboxymethylcellulose (CMC) and measuring glucose release at 540 nm (25). The glucose standard curve was y = 1.115x - 0.0193 (*R*^2^ = 0.9987, where y = absorbance at 540 nm, x = glucose in mg/mL) (Supplementary Figure S3e). One unit (U) of activity was defined as the enzyme amount producing 1 μmol of glucose per minute.

### 2.6 Microbial community analysis

Community genomic DNA was extracted using the E. Z. N. A. Soil DNA Kit (Omega, M5635-02, USA) following the manufacturer’s instructions (26). DNA concentration was measured with a Qubit fluorometer to ensure adequate yield. Sequencing libraries were prepared from 500 ng of DNA per sample using a library prep kit. The DNA was fragmented to approximately 500 bp, end-repaired, adapter-ligated, and PCR-amplified. Library quality was assessed with a Qubit 4.0, and paired-end sequencing was conducted on an Illumina NovaSeq 6000 platform. Raw reads underwent quality filtering with Fastp to remove adapters, trim low-quality bases (Q < 20) from the 3′ end using a 4-bp sliding window, correct inconsistent bases in overlapping paired reads, and discard reads shorter than 35 nt. Clean reads were assembled using Megahit for multi-sample mixed assembly, followed by SPAdes for reassembling unmapped reads. Binning was executed with MetaWRAP, including bin sorting, purification, quantification, reassembly, and identification to obtain high-integrity, low-contamination draft genomes. Gene prediction was performed with Prodigal (≥100 bp), and a non-redundant gene set was constructed using CD-HIT. Gene abundances were quantified using Salmon from the non-redundant set. Functional and taxonomic annotations were conducted by aligning sequences against KEGG, CAZy, and other databases using DIAMOND (E-value < 1e-5, Score > 60). KEGG Orthology (KO) entries were assigned based on the best-hit annotation. Pathway enrichment analysis was conducted by mapping KO entries to KEGG pathway maps. The relative abundance of each pathway was calculated as the ratio of reads assigned to that pathway to the total reads assigned to all KEGG pathways. Pathways were classified according to the KEGG hierarchical structure (Level 1, Level 2, and Level 3). Statistical comparison of pathway abundances between treatment groups was performed using the Metastats method, which calculates the abundance difference and significance based on the Fisher’s exact test with Benjamini-Hochberg FDR correction.

### 2.7 Untargeted metabolomics study by UPLC-MS technique

Samples were ground into a fine powder at 30 Hz for 30 s in liquid nitrogen. A 50 mg aliquot was extracted with 600 μL of 70% methanol containing an internal standard. This mixture underwent six vortexing cycles of 30 s each, was chilled at −20 °C for 30 min, and centrifuged at 12,000 rpm for 3 min. The supernatant was filtered through a 0.22 μm PTFE membrane filter (Jinteng Experiment Equipment Co., Ltd., Tianjin, China) and stored in vials for UPLC-MS analysis (27). HPLC separation occurred on a Waters HSS T3 column (2.1 × 100 mm, 1.8 μm) at 40 °C, with a flow rate of 0.40 mL/min and an injection volume of 4 μL. The mobile phases were water with 0.1% formic acid (A) and acetonitrile with 0.1% formic acid (B). The gradient program was: 0 min, 95% A/5% B; 2 min, 25% A/75% B; 4 min, 1% A/99% B (held for 1.5 min); then returned to 95% A/5% B within 0.1 min and held for 1.4 min. Mass spectrometry was conducted using a Q-Exactive system with electrospray ionization in both positive and negative ion modes. Full-scan MS acquisition covered m/z 84–1250 at a resolution of 35,000. Key settings included a spray voltage of 3.5 kV (positive) / 3.2 kV (negative), sheath gas at 30 arbs, auxiliary gas at 5 arbs, an ion transfer tube temperature of 320 °C, a vaporizer temperature of 300 °C, collision energies of 30/40/50 V. Top-10 data-dependent MS^2^ (tandem mass spectrometry) scans using higher-energy collisional dissociation (HCD) at normalized collision energies (NCE) of 30/40/50 V, with a dynamic exclusion duration of 3 s to prevent repeated sampling of the same precursor ion. Metabolite identification was achieved by matching accurate masses, fragmentation spectra, and retention times against public databases including PubChem, Metlin, KEGG compound and the Human Metabolome Database (HMDB). Significant differential metabolites were identified using variable importance in projection (VIP) scores > 1, combined with Student’s t-test (*p* < 0.05) and fold change (FC > 2 or FC < 0.5).

### 2.8 GC–MS analysis

A 30 g fresh sample was subjected to ultrasonic extraction with 75% ethanol at a 1:5 (w/v) ratio. The extraction conditions were set to 100 W and 40 °C for 1 h. After filtration, the solid residue was re-extracted for 30 min under identical conditions. The two filtrates were then combined and concentrated at 45 °C under reduced pressure. A 15 mL aliquot of this concentrate was saturated with sodium chloride and extracted three times with dichloromethane (3 × 15 mL). The combined organic layers were dried over anhydrous Na₂SO₄ overnight and spiked with 100 μL of 2,6-dichlorotoluene (10 mg/mL) as an internal standard. The extract was slowly evaporated to a final volume of 1.0 mL and filtered through a 0.22 μm organic filter. GC–MS analysis was conducted using a DB-5MS capillary column (60 m × 0.25 mm × 0.25 μm) with helium as the carrier gas at a flow rate of 1.0 mL/min (28). The injector temperature was set to 230 °C in split mode (10:1). The oven temperature program began at 40 °C for 2 min, increased to 230 °C at 3 °C/min, and held for 5 min, then rose to 280 °C at 5 °C/min with a 10-min hold. The mass spectrometer operated in electron ionization mode (70 eV), maintaining ion source and quadrupole temperatures at 230 °C and 150 °C, respectively. Data were recorded in full-scan mode over the m/z range of 35–500, with a solvent delay of 6 min. Compounds were identified by matching mass spectra against the NIST 17 database (match factor > 800) and by comparing retention indices (RIs) with NIST WebBook values. Three confidence levels were applied: (1) confirmed by mass spectrum and RI with an authentic standard; (2) matched by mass spectrum and RI; (3) matched by mass spectrum only. Semi-quantification used 2,6-dichlorotoluene as the internal standard, with concentrations calculated as (peak area ratio × IS concentration) / sample weight, expressed as μg/g dried sample. Peak detection, alignment, and deconvolution were performed on the Majorbio Cloud platform using the NIST MS Search algorithm across all 15 samples. Data were normalized to the internal standard and sample dry weight.

### 2.9 Statistical analysis

All experiments were conducted in triplicate with biological replicates. The data are shown as mean ± standard deviation. To evaluate mean differences, a one-way analysis of variance (ANOVA) was performed, followed by Tukey’s HSD test with a significance level of *p* < 0.05. Beta diversity, which assesses microbiome differences among samples, was analyzed using dimensionality-reduction techniques for visual representation. These analyses were visualized with the R vegan package (version 2.5–6). Metastats identified microorganisms or functions with significant differences between two groups, while LEfSe (version 1.1.0) analyzed differential microorganisms and functions across multiple groups.

## 3 Results and discussion

### 3.1 Physical and chemical properties after fermentation

Using raw *P. thomsonii* as the control (Y), we employed solid-state fermentation (SSF) to assess how various fermentation methods affect the nutritional components, isoflavonoid compounds, and amino acid composition of *P. thomsonii*. The methods compared were natural fermentation (K), bacterial fermentation with *B. velezensis* (B), yeast fermentation with *S. fibuligera* (S), and co-fermentation with both *B. velezensis* and *S. fibuligera* (M).

**Figure 1:**
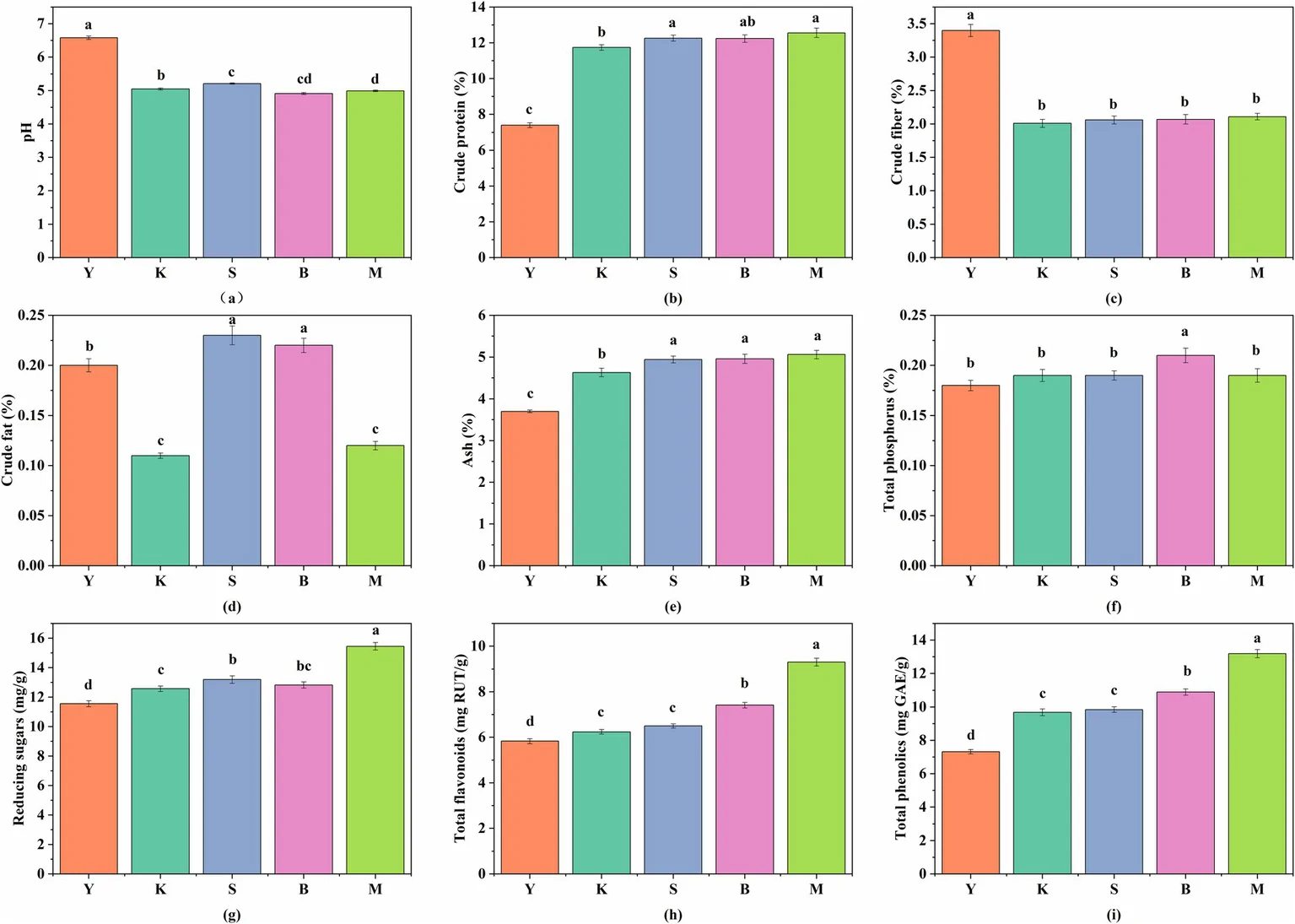
Nutritional analysis of fermented *P. thomsonii* (*n* = 3 biological replicates per group). **(a)** pH; **(b)** crude protein (%); **(c)** crude fiber (%); **(d)** crude fat (%); **(e)** ash (%); **(f)** total phosphorus (%); **(g)** reducing sugar (mg/g); **(h)** total flavonoid content (mg *RUT*/g); **(i)** total phenolic content (mg *GAE*/g). Data are presented as mean ± *SD*. Bars with different letters indicate significant differences (*p* < 0.05). Y, raw *P. thomsonii*; K, natural fermentation; S, yeast fermentation; B, bacterial fermentation; M, yeast–bacterial co-fermentation.

**Figure 2:**
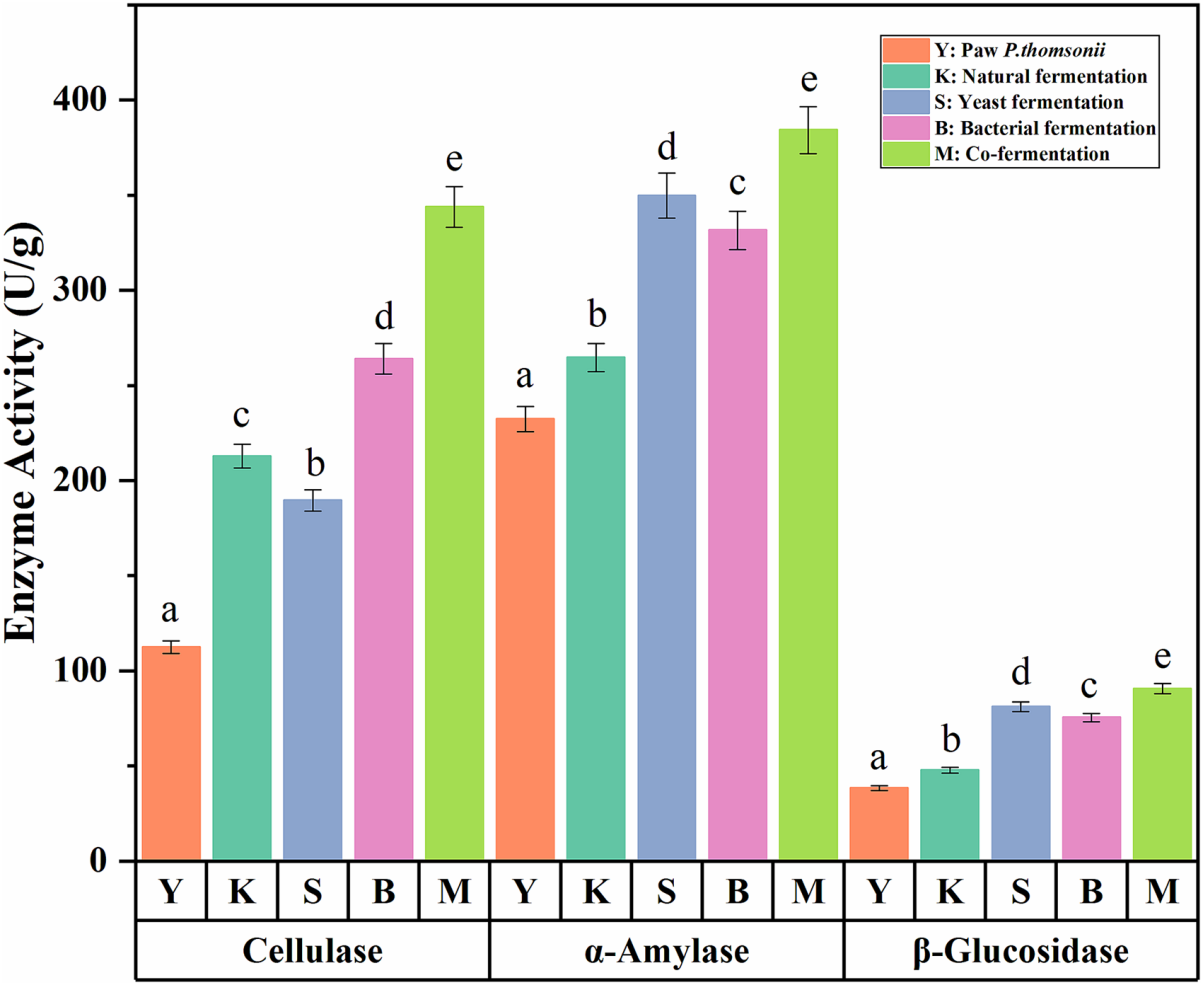
Enzyme activities of fermented *P. thomsonii* (*n* = 3 biological replicates per group). One unit (U) is defined as the enzyme amount producing 1 μmol of product (maltose, *p*-nitrophenol, or glucose, respectively) per minute under the assay conditions. Data are presented as mean ± SD. Bars with different letters indicate significant differences (*p* < 0.05). Y, raw *P. thomsonii*; K, natural fermentation; S, yeast fermentation; B, bacterial fermentation; M, yeast–bacterial co-fermentation.

**Figure 3:**
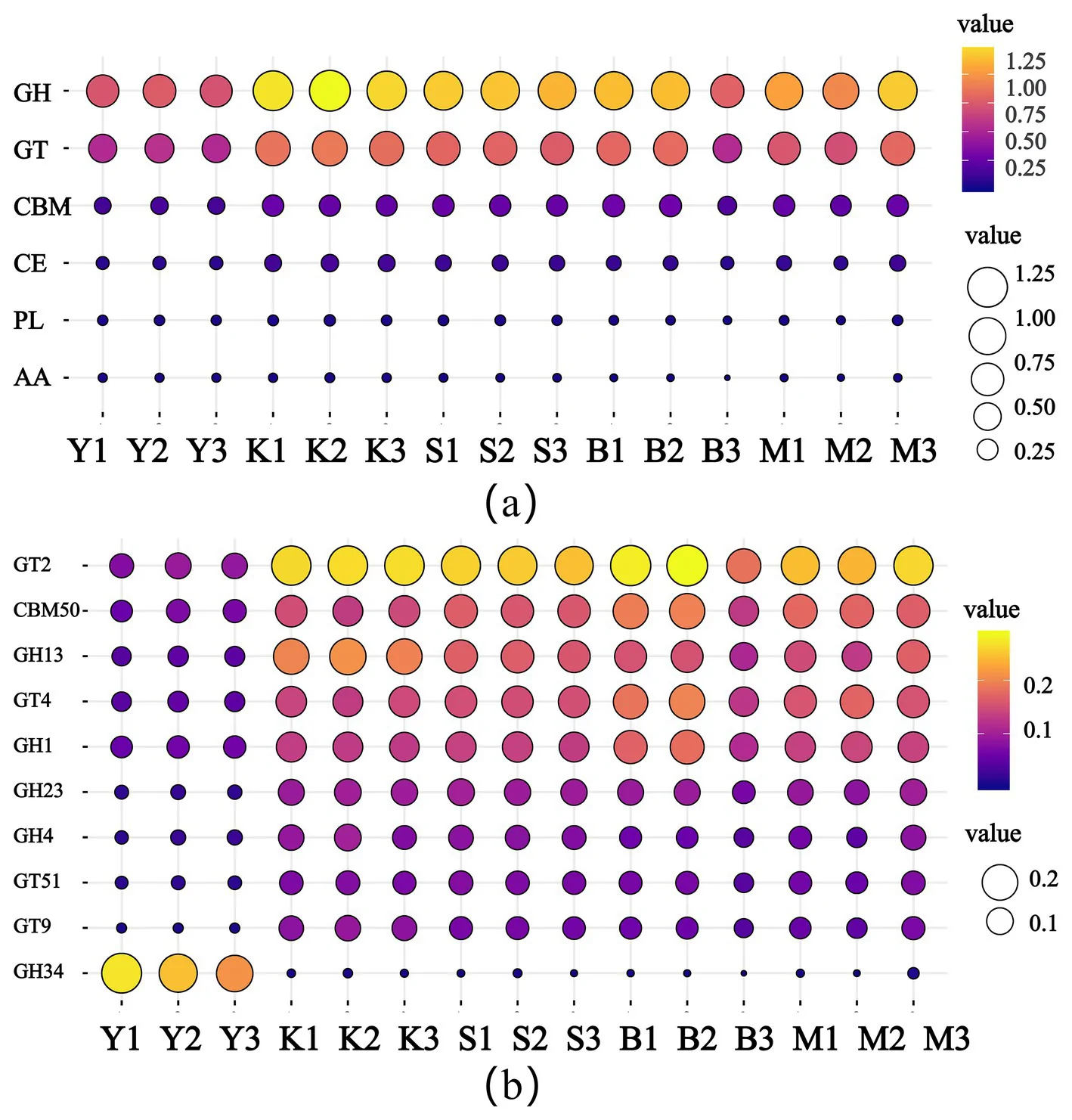
Relative abundance of carbohydrate-active enzyme (CAZy) functional genes during SSF of *P. thomsonii* (*n* = 3 biological replicates per group). **(a)** Stacked bar plot showing the relative abundance (%) of major CAZy classes across treatment groups: Glycoside hydrolases (GH), glycosyltransferases (GT), carbohydrate-binding modules (CBM), polysaccharide lyases (PL), and auxiliary activities (AA). Each bar represents one biological replicate. **(b)** Abundance profiles of core CAZy functional genes (GH1, GH13, CBM50, and other selected families) across treatment groups. Gene abundance was quantified from the non-redundant gene catalog using Salmon. Bars represent the mean of three biological replicates. Y, raw *P. thomsonii*; K, natural fermentation; S, yeast fermentation; B, bacterial fermentation; M, yeast–bacterial co-fermentation.

**Figure 4:**
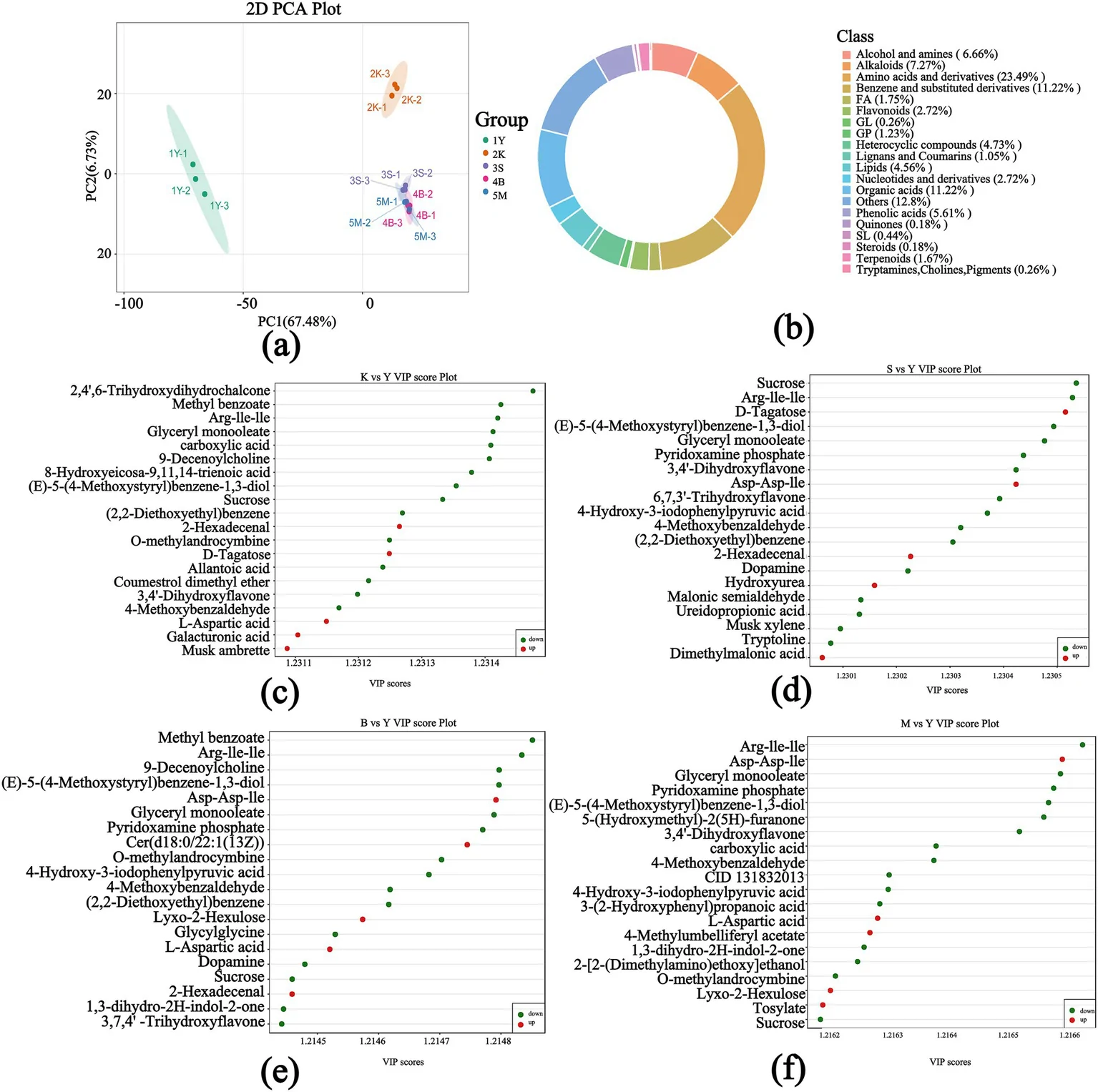
Metabolomics analysis of solid-state fermented *P. thomsonii* products (*n* = 3 biological replicates per group). **(a)** Two-dimensional principal component analysis (PCA) score plot showing the metabolic profiles of all 15 samples. PC1 and PC2 explained variance percentages are indicated on the axes. **(b)** Ring chart showing the relative abundance (%) of metabolite classes across all fermented samples. **(c–f)** Variable importance in projection (VIP) score plots identifying metabolites with VIP > 1.0 that significantly discriminate each fermented group from raw material: **(c)** K vs. Y; **(d)** S vs. Y; **(e)** B vs. Y; **(f)** M vs. Y. Red and green bars indicate upregulated and downregulated metabolites, respectively, in the fermented group relative to Y. VIP scores were derived from PLS-DA modeling. Significant differential metabolites were defined by VIP > 1, *p* < 0.05, and fold change > 2 or < 0.5. Y, raw *P. thomsonii*; K, natural fermentation; S, yeast fermentation; B, bacterial fermentation; M, yeast–bacterial co-fermentation.

Figure 1 illustrates how SSF significantly altered the nutritional and functional properties of *P. thomsonii*, with variations based on the fermentation strategy. The pH decreased notably in all fermented groups (Figure 1a), with the lowest value in S (4.91 ± 0.01), indicating microbial production of organic acids like lactic and acetic acids (29, 30). Post-fermentation, crude protein content increased significantly (Figure 1b), rising from 7.40 ± 0.13% in Y to between 11.74 ± 0.15% (K) and 12.56 ± 0.26% (M). This increase is attributed to microbial biomass accumulation, proteolysis with nitrogen retention, and *de novo* amino acid synthesis, with yeast and bacterial metabolism likely enhancing co-fermentation (31, 32). Crude fiber content (Figure 1c) initially at 3.40 ± 0.01% in Y, decreased significantly to 2.01–2.11% post-fermentation, indicating microbial degradation of structural polysaccharides. Reducing sugar content increased from 11.55 ± 0.21 mg/g in Y to 15.45 ± 0.26 mg/g in co-fermentation (Figure 1g). This rise is linked to the hydrolysis of starch and other polysaccharides by microbial *α*-amylase (Figure 2) (33). The highest accumulation in co-fermentation, despite moderate GH13 abundance (Figure 3), suggests a favorable balance between sugar production and consumption in co-fermentation (34). This disconnect between moderate GH13 gene abundance and highest reducing sugar accumulation may reflect post-transcriptional or post-translational regulation, whereby enzyme secretion efficiency and specific activity—rather than gene copy number—determine actual hydrolytic output (35). Significant changes were observed in TFC and TPC (Figures 1h,i): TFC increased from 5.83 ± 0.11 mg RUT/g in Y to 6.24 ± 0.10 (K), 6.50 ± 0.09 (B), 7.41 ± 0.12 (S), and 9.30 ± 0.17 (M) mg RUT/g, while TPC rose from 7.32 ± 0.13 mg GAE/g in Y to 9.68 ± 0.20 (K), 9.84 ± 0.17 (B), 10.89 ± 0.19 (S), and 13.19 ± 0.25 (M) mg GAE/g, with co-fermentation significantly outperforming all other treatments. This superior performance of co-fermentation is likely due to (i) microbial *β*-glucosidase activity (Figure 2) converting flavonoid glycosides to more extractable aglycones (36), (ii) yeast and bacteria enhancing cell wall-degrading enzymes (Figure 3) to release bound phenolics, and (iii) the release of bound phenolics and/or *de novo* synthesis of phenolic compounds detected in the metabolomics analysis (Figure 4).

Table 1 presents the amino acid contents (g/kg) of 17 amino acids and total amino acids (TAA) in Y and four fermented groups. Fermentation increased TAA from 44.16 ± 0.52 g/kg in Y to 49.04 ± 0.38 g/kg (K), 49.13 ± 0.41 g/kg (B), 50.94 ± 0.47 g/kg (S), and 52.11 ± 0.53 g/kg (M), mirroring the crude protein rise shown in Figure 1b. The highest TAA in co-fermentation suggests that yeast and bacteria together effectively enhance amino acid accumulation through microbial biomass, proteolysis, and de novo synthesis (37). For essential amino acids, lysine increased from 2.28 ± 0.03 g/kg in Y to 2.44–2.60 g/kg in the fermented groups, peaking in co-fermentation at 2.60 ± 0.02 g/kg. Phenylalanine decreased from 2.48 ± 0.01 g/kg in Y to 1.69–1.83 g/kg, likely due to its role as a precursor in flavonoid synthesis (38). Methionine, absent in Y, appeared in all fermented groups, with co-fermentation showing the highest level (0.44 ± 0.01 g/kg), indicating microbial synthesis of this sulfur-containing amino acid, typically scarce in plants (39). Threonine, isoleucine, and leucine increased post-fermentation, with leucine showing the largest rise (from 2.08 ± 0.02 g/kg in Y to 3.08 ± 0.03 g/kg in co-fermentation). Valine and histidine slightly decreased, potentially reducing the risk of biogenic amine formation (40). Overall, fermentation balanced the amino acid profile, aligning it closer to an ideal protein (41). Regarding flavor-related amino acids, aspartic acid increased from 11.53 ± 0.27 g/kg in Y to 16.13 ± 0.27 g/kg in co-fermentation. Glutamic acid rose from 4.60 ± 0.11 g/kg in Y to 6.79 ± 0.09 g/kg in B, likely due to active bacterial glutamate synthesis (42). Alanine increased from 2.14 ± 0.01 to 3.22 ± 0.02 g/kg in S, and glycine rose from 1.54 ± 0.02 to 2.11 ± 0.02 g/kg in S. Proline decreased from 6.04 ± 0.05 to 4.44–5.22 g/kg, with the lowest in B. Thus, fermentation enhanced umami and sweet amino acids, improving taste, with co-fermentation excelling in umami amino acids. Serine slightly increased from 1.82 ± 0.01 g/kg in Y to 1.98 ± 0.02 g/kg in co-fermentation. Arginine decreased from 1.34 ± 0.01 g/kg in Y to 1.15–1.26 g/kg, lowest in B, consistent with microbial conversion to ornithine or citrulline. Tyrosine sharply decreased from 1.30 ± 0.01 g/kg in Y to 0.66–0.79 g/kg, lowest in B, supporting its conversion to dopamine (detected in S, Figure 4) and other metabolites. In summary, SSF, especially co-fermentation, significantly improved the amino acid composition of *P. thomsonii*, increasing TAA by about 18%, balancing essential amino acids, and enhancing umami and sweet amino acids.

**Table 1:** Amino acid composition of fermented *P. thomsonii* (g/kg)^*^.

Items (g/kg)	Y group	K group	B group	S group	M group
Lysine	2.28 ± 0.03^a^	2.48 ± 0.01^b^	2.45 ± 0.01^b^	2.44 ± 0.02^b^	2.60 ± 0.02^c^
Phenylalanine	2.48 ± 0.01^d^	1.73 ± 0.01^a^	1.69 ± 0.01^a^	1.78 ± 0.02^b^	1.83 ± 0.01^c^
Methionine	ND	0.42 ± 0.01^b^	0.41 ± 0.01^ab^	0.41 ± 0.01^a^	0.44 ± 0.01^c^
Threonine	1.52 ± 0.02^a^	1.66 ± 0.03^b^	1.69 ± 0.03^b^	1.78 ± 0.02^c^	1.81 ± 0.03^c^
Isoleucine	1.51 ± 0.03^a^	1.56 ± 0.02^ab^	1.60 ± 0.02^b^	1.67 ± 0.01^c^	1.72 ± 0.01^c^
Leucine	2.08 ± 0.02^a^	2.80 ± 0.02^b^	2.88 ± 0.03^c^	2.98 ± 0.03^d^	3.08 ± 0.03^e^
Valine	2.52 ± 0.02^d^	2.36 ± 0.02^b^	2.35 ± 0.02^a^	2.44 ± 0.03^c^	2.49 ± 0.03^d^
Histidine	1.46 ± 0.03^d^	1.35 ± 0.01^c^	1.28 ± 0.01^a^	1.32 ± 0.01^b^	1.37 ± 0.02^c^
Aspartic acid	11.53 ± 0.27^a^	15.30 ± 0.23^b^	14.81 ± 0.19^b^	15.42 ± 0.36^b^	16.13 ± 0.27^c^
Glutamic acid	4.60 ± 0.11^a^	5.62 ± 0.07^b^	6.79 ± 0.09^d^	6.57 ± 0.11^cd^	6.52 ± 0.08^c^
Serine	1.82 ± 0.01^a^	1.85 ± 0.03^ab^	1.89 ± 0.02b^c^	1.94 ± 0.02^c^	1.98 ± 0.02^d^
Proline	6.04 ± 0.05^d^	5.22 ± 0.02^c^	4.44 ± 0.07^a^	4.92 ± 0.05^b^	4.98 ± 0.07^b^
Glycine	1.54 ± 0.02^a^	1.87 ± 0.01^b^	1.91 ± 0.01^b^	2.11 ± 0.02^d^	1.98 ± 0.02^c^
Arginine	1.34 ± 0.01^d^	1.20 ± 0.01^b^	1.15 ± 0.01^a^	1.20 ± 0.01^b^	1.26 ± 0.01^c^
Alanine	2.14 ± 0.01^a^	2.86 ± 0.03^b^	3.13 ± 0.02^c^	3.22 ± 0.02^d^	3.13 ± 0.03^c^
Tyrosine	1.30 ± 0.01^d^	0.76 ± 0.01^b^	0.66 ± 0.01^a^	0.74 ± 0.01^b^	0.79 ± 0.01^c^
Total amino acids	44.16 ± 0.52^a^	49.04 ± 0.38^b^	49.13 ± 0.41^b^	50.94 ± 0.47^b^	52.11 ± 0.53^c^

^*^Values are presented as mean ± standard deviation (n = 3 biological replicates per group). Different superscript letters within each row indicate significant differences (*p* < 0.05). ND, not detected. Y, raw *P. thomsonii*; K, natural fermentation; S, yeast fermentation; B, bacterial fermentation; M, yeast-bacterial co-fermentation. Amino acid quantification was performed according to GB/T 18246–2019.

Nutritional enhancements are directly linked to the enzymatic functions of fermenting microorganisms. Key carbohydrase and hydrolase enzymes are crucial for breaking down polysaccharides, releasing bound phenolics, and synthesizing amino acids and other metabolites. The next section examines the activities of three essential carbohydrate-active enzymes to elucidate the enzymatic basis for the observed quality improvements.

### 3.2 Enzyme activities after fermentation

**Figure 5:**
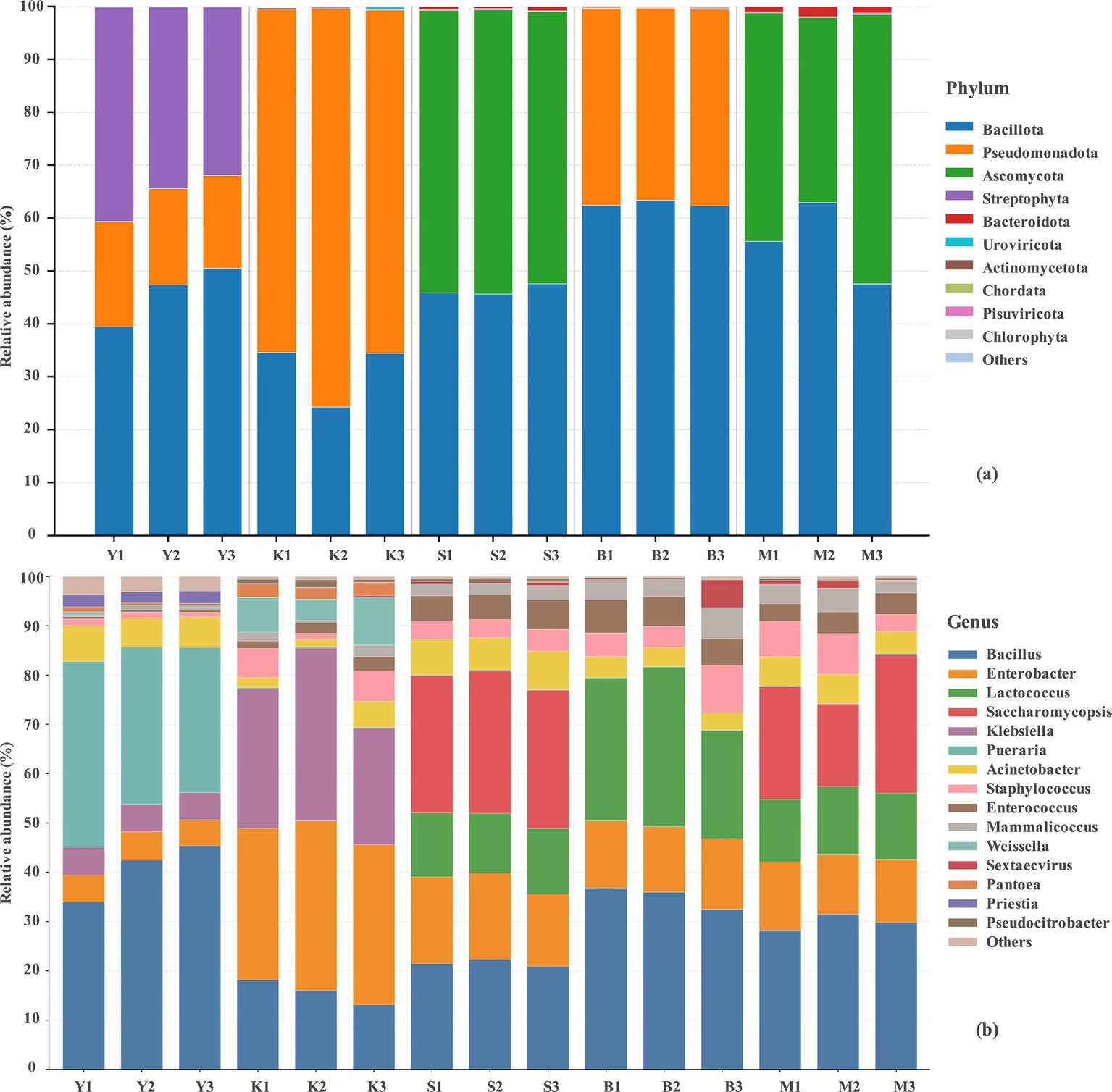
Microbial community structure during SSF of *P. thomsonii* (*n* = 3 biological replicates per group). **(a)** Relative abundance (%) of microbial taxa at the phylum level. Each bar represents one biological replicate. Taxa with relative abundance < 1% were grouped as “Others”. **(b)** Relative abundance (%) of microbial taxa at the genus level. Each bar represents one biological replicate. The top 15 most abundant genera are shown; remaining genera were grouped as “Others.” Taxonomic assignment was based on GTDB-Tk classification of metagenomic sequences. Y, raw *P. thomsonii*; K, natural fermentation; S, yeast fermentation; B, bacterial fermentation; M, yeast–bacterial co-fermentation.

Figure 2 presents enzyme activities (U/g) of *α*-amylase, *β*-glucosidase, and cellulase in Y and the four fermented groups: K, S, B, and M. α-Amylase activity increased from 232.32 ± 6.52 U/g in Y to 264.71 ± 7.39 (K), 349.77 ± 11.72 (S), 331.51 ± 10.02 (B), and 384.21 ± 12.31 (M). The higher activities in S and B align with the presence of amylase (GH13 family) in single-strain fermentations (Figure 3). The highest reducing sugar content in co-fermentation (15.45 ± 0.26 mg/g; Figure 1) suggests co-fermentation utilizes additional enzymes for efficient sugar production and retention (43). *β*-Glucosidase activity rose from 38.29 ± 1.29 U/g in Y to 47.81 ± 1.51 (K), 81.14 ± 2.57 (S), 75.43 ± 2.15 (B), and 90.67 ± 2.66 (M), with co-fermentation showing the highest activity. This enzyme converts cellobiose to glucose and hydrolyzes flavonoid glycosides to aglycones, consistent with the highest TFC and TPC in M and its GH1/GH3 abundance (Figure 3). Cellulase activity increased from 112.38 ± 3.29 U/g in Y to 212.83 ± 6.31 (K), 189.51 ± 5.59 (S), 263.94 ± 8.02 (B), and 343.77 ± 10.75 (M). High activity in co-fermentation and B corresponds with increased GH families and CBM modules (Figure 3). Microbial community analysis (Figure 5) indicates that the yeast-dominated S group had high *α*-amylase but moderate cellulase activity, while the bacterium-dominated B group consistently exhibited high activity across all enzymes. co-fermentation demonstrated the highest β-glucosidase and cellulase activities. The CAZy gene abundance profiles (Figure 3) support these findings. Overall, the highest β-glucosidase activity in co-fermentation correlates with its superior release of flavonoid aglycones and phenolics. In contrast, the balanced α-amylase and cellulase activities in co-fermentation provide ample reducing sugars (Figure 1) as carbon sources and precursors, enhancing the functional and flavor benefits of co-fermentation (44).

The enzyme activity data prompted an investigation into the genomic basis of these capabilities. Metagenomic sequencing was used to examine the taxonomic composition, functional gene repertoire, and carbohydrate-active enzyme (CAZy) profiles linked to the observed enzymatic activities (see Section 3.3.3).

### 3.3 Microbial community composition after fermentation

#### 3.3.1 Metagenomic sequencing overview and gene catalog

Metagenomic sequencing of the five treatment groups (Y, K, S, B, and M; n = 3 per group) yielded approximately 659 million paired-end reads, each 150 bp long. Across all 15 samples, N50 values ranged from 787 bp (Y) to 2,060 bp (K), with a mean of 1,642 bp. The total assembly length ranged from 67.9 Mb (M) to 196.0 Mb (Y). The Y group exhibited notably higher contig counts (154,298–226,461) but lower N50 values (787–810 bp), likely reflecting the higher complexity of the raw plant-associated microbiome. The fermented groups (K, S, B, M) showed more moderate contig counts (45,640–65,675) with higher N50 values (1,609–2,060 bp), consistent with the expected reduction in microbial diversity following fermentation. The non-redundant gene catalog comprised 516,233 unigenes with an N50 of 612 bp, total length of 240,260,046 bp, and GC content of 45.98%. Our pipeline included CheckM2 v1.0.1 for quality assessment of microbial genome bins, with MetaWRAP v1.3.2 used for bin refinement and reassembly, and dRep v2.6.2 for genome de-replication and selection of representative genomes. CoverM v0.6.1 was used for coverage estimation of metagenomic assemblies. The mean clean reads per sample was 41,701,155, with a standard deviation of 2,358,964 and a coefficient of variation (CV) of 5.66%. This low CV indicates generally even sequencing depth across samples. The Y group exhibited slightly lower reads (mean 38,481,126 per sample), which can be attributed to higher host (plant) genome contamination in the raw material—host-derived reads accounted for 2.048% of reads in Y versus 0.3–0.4% in fermented samples. After quality filtering with Fastp and host decontamination, clean read retention rates ranged from 93.50 to 96.51%.

#### 3.3.2 Taxonomic composition and community reorganization

At the phylum level (Figure 5a), Bacillota (syn. Firmicutes) consistently dominated, ranging from 44.17 ± 8.45% in Y to 62.69 ± 0.57% in B. This phylum maintained high abundance across all groups, particularly enriched in B and co-fermentation, demonstrating its adaptability to fermentation environments. Streptophyta was notably enriched in Y (35.55 ± 4.48%) but constituted only 0.13–0.30% in fermentation groups, indicating that plant-derived DNA in the raw substrate is effectively degraded during fermentation. Pseudomonadota exhibited a polarized pattern. Under natural fermentation (K), it surged to 68.36 ± 6.02%, becoming the dominant group, but was severely suppressed (< 0.01%) in S and co-fermentation. In B, it maintained moderate abundance (36.90 ± 0.49%). These results suggest that yeast presence strongly inhibits Pseudomonadota. Competitive exclusion via resource depletion is the dominant mechanism, with antimicrobial volatile organic compounds (VOCs, Table 2) production and pH reduction (Figure 1) serving as supplementary mechanisms, consistent with the broader fermentation ecology literature recognizing that yeast-mediated microbial suppression typically involves both niche competition and antimicrobial metabolite production (45, 46). Ascomycota enrichment is a hallmark of yeast fermentation. In S (52.85 ± 1.22%) and co-fermentation (43.03 ± 7.97%), Ascomycota was significantly enriched, while it was nearly undetectable in K and B, indicating that Ascomycota abundance directly reflects yeast fermentation extent (47). Each fermentation mode formed distinct microbial community structures, with co-fermentation showing characteristics of both yeast and bacterial fermentation.

**Table 2:** Top Spearman rank correlations between metagenomic genera and isoflavone aglycone-related metabolites (|ρ| ≥ 0.7, FDR < 0.05)^*^.

Genus	Metabolite	*ρ* (rho)	*p*-value	FDR	Direction
*Citrobacter*	Hispidol	0.961	<0.001	0.000004	Positive
*Bacillus*	Hispidol	−0.957	<0.001	0.000004	Negative
*Enterobacter*	Hispidol	0.896	<0.001	0.0004	Positive
*Enterococcus*	Naringenin chalcone	−0.896	<0.001	0.0004	Negative
*Citrobacter*	7,8,4′-Trihydroxyisoflavone	0.854	<0.001	0.001	Positive
*Bacillus*	7,8,4′-Trihydroxyisoflavone	−0.836	<0.001	0.002	Negative
*Enterobacter*	7,8,4′-Trihydroxyisoflavone	0.829	<0.001	0.002	Positive
*Mammaliicoccus*	Naringenin chalcone	−0.821	<0.001	0.002	Negative
*Klebsiella*	Naringenin chalcone	0.821	<0.001	0.002	Positive
*Lactococcus*	Naringenin chalcone	−0.771	<0.001	0.006	Negative
*Serratia*	Naringenin chalcone	0.768	<0.001	0.007	Positive
*Pueraria*	Naringenin chalcone	0.739	0.002	0.012	Positive
*Streptococcus*	Naringenin chalcone	−0.721	0.002	0.015	Negative
*Weissella*	Naringenin chalcone	0.707	0.003	0.019	Positive

^*^Per-sample genus abundance values (%) were derived from GTDB-Tk-classified metagenomic taxonomy. Per-sample metabolite intensities were extracted from untargeted LC–MS/MS metabolomics. Spearman rank correlation coefficients (ρ) and two-tailed *p*-values were computed across all 15 biological samples (3 replicates × 5 groups). Benjamini–Hochberg false discovery rate (FDR) correction was applied for multiple testing. Only correlations with |*ρ*| ≥ 0.7 and FDR < 0.05 are shown. “Positive” and “Negative” indicate the direction of correlation. Y, raw *P. thomsonii*; K, natural fermentation; S, yeast fermentation; B, bacterial fermentation; M, yeast-bacterial co-fermentation.

At the genus level (Figure 5b), *Bacillus* dominated various treatment groups, demonstrating its adaptability to fermentation environments and stability within the indigenous microbial community of the raw materials. Significant differences in *Bacillus* abundance appeared across fermentation methods (*p* < 0.05). The highest abundances were in B (35.08 ± 2.29%) and S (21.57 ± 0.69%), while lower levels occurred in K (15.75 ± 2.52%) and co-fermentation (29.84 ± 1.62%). Plant-derived genera like *Pueraria* and *Glycine*, initially abundant in raw materials (33.01 ± 4.19% and 2.23 ± 0.26%, respectively), dropped to below 0.2% in all fermentation groups. This reduction indicates the fermentation process effectively removed plant DNA signals, consistent with phylum-level changes in Streptophyta, marking fermentation initiation. *Enterobacter* and *Klebsiella* exhibited rapid proliferation during natural fermentation, dominating in K with a combined abundance of 61.52%. This suggests avoiding uncontrolled natural fermentation in food industry applications (48). The dominance of these opportunistic pathogens in K underscores the biosafety advantage of using defined starter cultures (49, 50). However, these genera were significantly suppressed in S and co-fermentation, aligning with phylum-level trends in Pseudomonadota, indicating that yeast presence strongly inhibits *Enterobacteriaceae*. *Saccharomycopsis*, the inoculated yeast genus, was significantly enriched in S (28.27 ± 0.52%) and co-fermentation (22.55 ± 5.64%) but undetectable in K and B. Its abundance reflects the extent of yeast fermentation and distinguishes it from other fermentation modes. Although the lower abundance of *Saccharomycopsis* in co-fermentation compared to S was not statistically significant (*p* > 0.05), the biological trend is consistent with competitive niche partitioning with *Bacillus* or resource reallocation toward secondary metabolism rather than biomass accumulation. *Lactococcus* was significantly enriched in B and co-fermentation, with the highest abundance in B (27.76 ± 5.36%) and notable levels in co-fermentation (13.31 ± 0.60%). It was virtually undetectable in Y and K, indicating active lactic acid bacteria metabolism (51). *Weissella* was specifically enriched in K (7.03 ± 2.70%) and nearly absent in other fermentation groups (< 0.02%).

#### 3.3.3 Analysis of functional annotations

**Figure 6:**
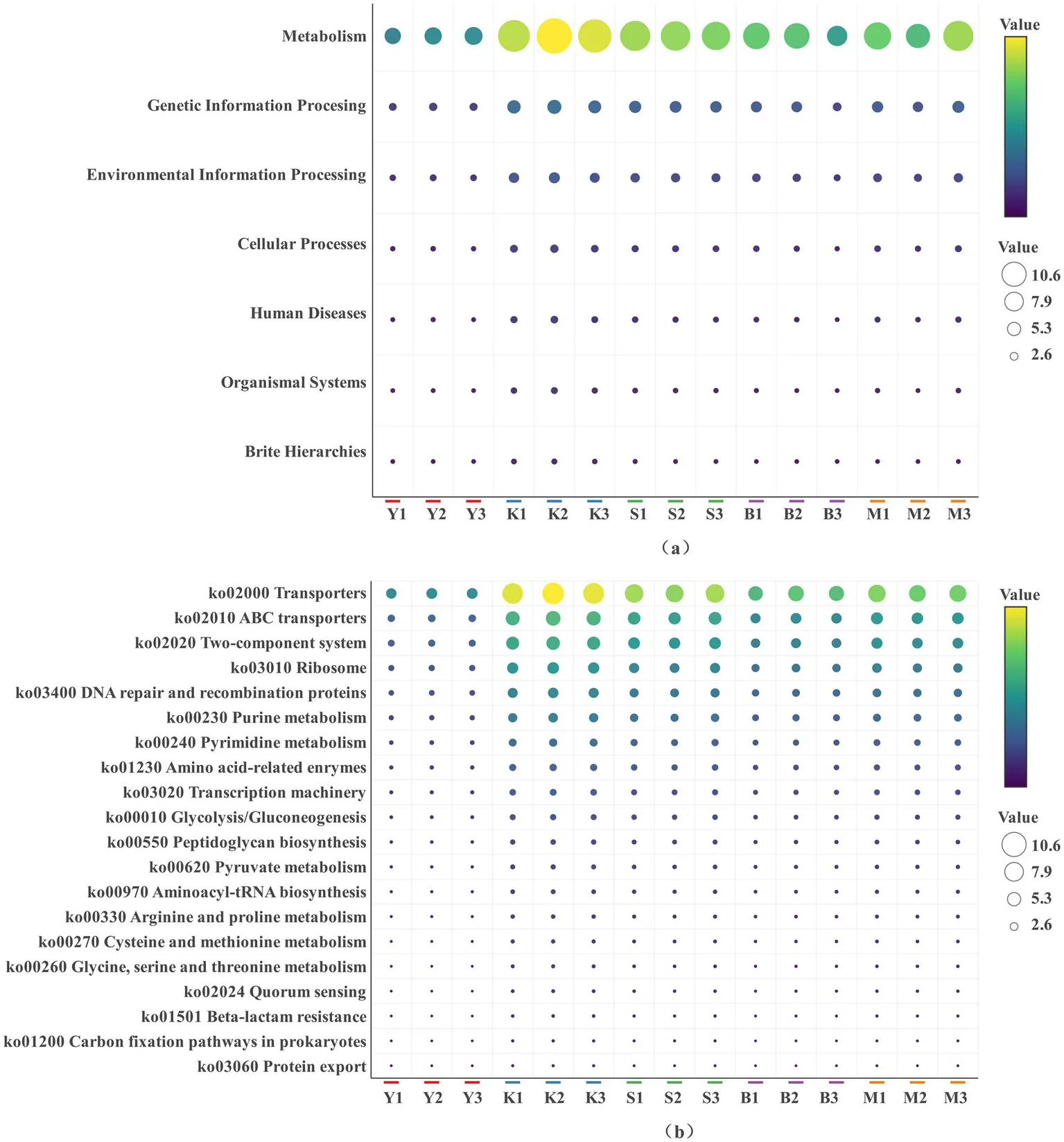
KEGG functional annotation of metagenomic sequences during SSF of *P. thomsonii* (*n* = 3 biological replicates per group). **(a)** Relative abundance (%) of KEGG Level 1 functional categories across treatment groups. The five Level 1 categories are: Cellular Processes, Environmental Information Processing, Genetic Information Processing, Metabolism, and Organismal Systems. **(b)** Relative abundance (%) of the top 20 KEGG Level 3 pathways. Pathway abundance was calculated as the proportion of reads annotated to each pathway relative to total KEGG-annotated reads per sample. Bars represent the mean of three biological replicates. Y, raw *P. thomsonii*; K, natural fermentation; S, yeast fermentation; B, bacterial fermentation; M, yeast–bacterial co-fermentation.

The KEGG annotation identified 7,737 KO entries and 521 metabolic pathways. At Level 1 (Figure 6a), the Metabolism category (6–17%) was of particular interest, as it reflects the microbial metabolic capacity for substrate conversion. Category K exhibited the highest Metabolism abundance (15.91–16.91%), consistent with the diverse metabolic activity of the natural fermentation microbiota. At Level 3 (Figure 6b), the ko02000 pathway (Transporters) was most prevalent, suggesting high nutrient uptake in active fermenting communities (52). Among fermentation-specific pathways, glycolysis/gluconeogenesis (ko00010), pyruvate metabolism (ko00620), and peptidoglycan biosynthesis (ko00550) were significantly enriched in K, S, B, and M compared to Y. Notably, co-fermentation showed enrichment in both carbohydrate and amino acid metabolism, consistent with its higher reducing sugar accumulation and total amino acid content.

Figure 3 displays the relative abundances (measured at the DNA level) of major carbohydrate-active enzyme (CAZy) families—glycoside hydrolases (GH), glycosyltransferases (GT), carbohydrate-binding modules (CBM), polysaccharide lyases (PL), and auxiliary activity (AA) (53)—in Y and four fermentation treatments (K, S, B, and M), each with three biological replicates. The CAZy profile reveals the microbial capacity to degrade plant cell wall polysaccharides, indicating substrate conversion efficiency during SSF (54). CAZy annotation (Figure 3a) identified glycoside hydrolases (GH; 0.74–1.26%) as the dominant enzyme class, followed by glycosyltransferases (GT; 0.53–0.86%) and carbohydrate-binding modules (CBM; 0.14–0.29%). B and co-fermentation treatments showed elevated GH gene counts, aligning with the high cellulase and *β*-glucosidase activities observed (Figure 2), thus supporting the enzymatic data. In Y, the relative abundances of most CAZy families were low and consistent across replicates, indicating limited carbohydrate-degrading capacity of the microorganisms naturally present on the raw material. This suggests a weak initial degradation potential for macromolecular polysaccharides. All fermentation groups (K, S, B, M) exhibited significantly increased abundances of multiple CAZy families compared to Y, with B and co-fermentation showing strong enrichment of core functional genes like GH13, GH1, and CBM50 (Figure 3b). This enhancement of microbial glycoside hydrolase capacity facilitates the breakdown of complex polysaccharides in *P. thomsonii*, providing carbon sources for subsequent metabolism. GH13, a core functional gene for starch degradation, largely determines microbial utilization efficiency of starch substrates and drives carbon supply during fermentation. The genes can be resolved into 21 distinct sub-activities (e.g., *α*-amylase EC 3.2.1.1, α-glucosidase EC 3.2.1.20, isoamylase EC 3.2.1.68, neopullulanase EC 3.2.1.135, cyclomaltodextrin glucanotransferase EC 2.4.1.19). Its high abundance generally corresponds to increased substrate degradation rates and fermentation activity (55). GH1 is involved in degrading cell wall polysaccharides like cellulose and converting flavonoid glycosides, releasing aglycones (56). It should be noted that GH1 encompasses enzymes with diverse substrate specificities beyond *β*-glucosidase, including β-glucosidase (EC 3.2.1.21), β-galactosidase (EC 3.2.1.23), β-xylosidase (EC 3.2.1.37), β-mannosidase (EC 3.2.1.25), phlorizin hydrolase (EC 3.2.1.62), and 21 additional activities—precluding substrate-specific resolution at the family level. Therefore, gene-level abundance reflects the total hydrolytic potential of this family rather than β-glucosidase activity alone. Thus, it is a critical functional gene influencing both the pharmacological activity and flavor quality of fermented *P. thomsonii* products. In S, GH and GT families (e.g., GH1, GH13) were abundant, while PL and AA families were relatively low. This is consistent with yeast primarily utilizing mono- and oligosaccharides and relying on enzymes like invertase and maltase, with less need for pectin lyases and lignin-modifying enzymes. B exhibited a comprehensive repertoire of core functional genes, suggesting that bacteria possess a more complete polysaccharide-degrading enzyme system. Notably, the abundance of certain amylases (GH13) in B was lower than in S, indicating that bacteria may prefer other carbon sources under the tested conditions. K showed relatively high abundances across almost all CAZy families, suggesting that natural fermentation enriches a variety of functionally diverse microorganisms that secrete a broad range of CAZy enzymes, potentially enabling efficient substrate degradation. However, this also led to noticeable fluctuations in functional gene abundances between batches, reflecting community functional instability. Of particular interest, the abundance of AA family core genes in co-fermentation was significantly higher than in S but slightly lower than in B, with a broader range of variation. This pattern suggests that when yeast and bacteria coexist, they may promote the production of auxiliary enzymes through cross-feeding or other metabolic interactions, thereby degrading complex carbohydrates (57).

The microbial community structure and CAZy gene profiles provide the enzymatic foundation for substrate degradation and biotransformation. To assess the metabolic outcomes of these microbial activities, we performed untargeted metabolomics. This method enabled a comprehensive analysis of chemical composition changes resulting from different fermentation strategies.

### 3.4 Metabolomic profile after fermentation

Untargeted metabolomics using UPLC–MS in both positive and negative ionization modes identified a total of 1,693 metabolites across all samples (Level 2 accounted for 26.3% (446 metabolites); Level 3 accounted for 73.7% (1,247 metabolites)). Detailed m/z, retention time, and MS/MS match scores for differential metabolites are provided in Supplementary Table S2. These metabolites encompassed various chemical classes, such as organic acids, amino acids and derivatives, flavonoids (including isoflavones), lipids (like lysophospholipids), phenolic acids, alkaloids, heterocyclic compounds, saccharides, and lignans/coumarins. Figure 4a presents a two-dimensional principal component analysis (PCA) score plot for samples from different fermentation treatments. PC1 explains 67.48% of the total variance, while PC2 accounts for 6.73%. Y samples cluster tightly on the negative side of PC1, whereas all fermented groups (K, S, B, M) are distinctly separated on the positive side. This separation indicates that fermentation significantly altered the metabolome of *P. thomsonii*, showing greater chemical differences between raw and fermented products than among individual fermentation methods. The ring chart in Figure 4b displays the relative percentages of metabolite classes in fermented *P. thomsonii*. Amino acids and derivatives are the most abundant, comprising 23.49%, which suggests active proteolysis and amino acid metabolism during SSF, aligning with increased TAA (Table 1). Benzene and substituted derivatives, along with organic acids, each make up 11.22%, highlighting the importance of aromatic compounds and organic acid metabolism. Aromatic compounds are related to phenolic acids and flavonoid precursors, while organic acids reflect microbial metabolism (58, 59). Alkaloids constitute 7.27%, alcohols and amines 6.66%, phenolic acids 5.61%, heterocyclic compounds 4.73%, and lipids 4.56%. Flavonoids account for 2.72%, and lignans and coumarins for 1.05%. Although these are less abundant overall, they are enriched in co-fermentation, contributing to functional bioactivity, as shown by TFC and TPC in Figure 1. The “other” category, at 12.8%, includes unidentified or low-abundance components. Overall, Figure 4b reveals that the metabolome of fermented *P. thomsonii* is chemically diverse, dominated by amino acid derivatives, aromatic compounds, and organic acids. This diversity provides a metabolic basis consistent with the nutritional, flavor, and functional enhancements observed in earlier figures and tables.

The variable importance in projection (VIP) score plots (K vs. Y, S vs. Y, B vs. Y, and M vs. Y) revealed significant alterations in the metabolic profile of *P. thomsonii* due to all fermentation treatments compared to Y. VIP values over 1.0 indicate major contributions to group separation (Figures 4c–f). Generally, all four fermentation types primarily downregulated numerous metabolites, particularly long-chain fatty acid esters and aromatic derivatives, suggesting extensive microbial metabolism or degradation of these precursors. Concurrently, each treatment also upregulated certain metabolites, highlighting the synthetic and transformative roles of microorganisms during fermentation. Specifically, K exhibited the fewest differentially expressed metabolites, with only minor fluctuations in a few compounds. This suggests that the indigenous microbial community has relatively weak metabolic activity and exerts the least impact on the metabolic profile (K vs. Y, Figure 4c). S showed a more concentrated upregulation pattern, significantly elevating substances like 2-hexadecenal and D-tagatose, while downregulating certain sugars and aromatics. This underscores the specificity of yeast in sugar metabolism and flavor-precursor conversion (S vs. Y, Figure 4d). B displayed a wider variety of upregulated metabolites, including markedly increased levels of 2-hexadecenal and Asp-Asp-Ile. The downregulated metabolites generally had higher VIP scores, suggesting that bacterial fermentation induces more intense metabolic transformations of lipids, peptides, and fatty acids (B vs. Y, Figure 4e). Co-fermentation exhibited the greatest number of differentially expressed metabolites and the most pronounced upregulation among the four groups. Peptides such as Asp-Asp-Ile and Arg-Ile-Ile were strongly elevated, while many fatty acids and isoflavones were downregulated. This reflects the metabolic effects in the co-fermentation system, which not only enhances macromolecule degradation but also promotes the synthesis of small molecules and characteristic peptides, making it the fermentation method with the most substantial impact on the metabolome (M vs. Y, Figure 4f).

The metabolite profiles reveal significant biochemical pathways. The presence of galacturonic acid indicates pectin degradation, while the accumulation of flavonoids and phenolic compounds suggests an enhanced release of bound phenolics (60). The detection of dopamine, tryptamine, and pyridoxamine phosphate indicates active aromatic amino acid decarboxylation pathways (61). Multiple hydroxylated flavones in S support the idea that yeast effectively releases or modifies flavonoids (62). In B, the simultaneous presence of ceramide and trihydroxyflavone suggests active lipid metabolism and flavonoid hydroxylation (63). Notably, Garcinone C and indole-3-propionic acid were significantly enriched in co-fermentation, indicating active xanthone bioconversion and tryptophan-derived indole metabolism, consistent with elevated citric acid levels reflecting enhanced TCA cycle activity. Additionally, 4-(hydroxymethyl) pyrimidine and 4′-methoxychalcone further distinguish co-fermentation from other treatments. Overall, different fermentation methods achieve targeted alterations in *P. thomsonii* metabolites through distinct pathway regulation: co-fermentation shows the strongest metabolic regulatory capacity; single-strain fermentation uniquely upregulates specific metabolites; and natural fermentation maintains basic component stability through mild metabolic changes.

### 3.5 Correlating specific genera with aglycone production and ester formation

Per-sample genus abundance values (%) were extracted from the GTDB-Tk-classified metagenomic taxonomy (870 genera). Per-sample metabolite intensities were extracted from the untargeted LC-MS/MS metabolomics significant metabolites summary. Spearman rank correlation coefficients (rho) and two-tailed *p*-values were computed across all 15 biological samples (3 replicates × 5 groups). The analysis targeted 16 key fermentative genera against 22 metabolites categorized as isoflavone aglycones (3), ester/acid compounds (11), and flavor/aroma-related metabolites (8). A total of 352 pairwise correlations were computed, yielding 145 significant correlations (*p* < 0.05), of which 90 were highly significant (*p* < 0.01) and 61 showed strong correlations (|*ρ*| > 0.7). Benjamini-Hochberg false discovery rate (FDR) correction was applied to account for multiple testing, with 104 correlations surviving at q < 0.05, confirming the robustness of the key findings.

For aglycone production (Table 2), *Enterobacteriaceae* showed strong positive correlations with isoflavone aglycones: *Citrobacter* correlated with Hispidol (ρ = 0.961, FDR = 4 × 10^−6^) and 7,8,4′-trihydroxyisoflavone (ρ = 0.854, FDR = 0.001), and *Enterobacter* showed similar positive associations (ρ = 0.896 and 0.829, respectively). Conversely, *Bacillus* exhibited strong negative correlations with both aglycones (ρ = −0.957 and −0.836), reflecting competitive exclusion of *Enterobacteriaceae* and consequent reduction of *β*-glucosidase (GH1)-mediated deglycosylation.

For ester-related metabolism (Table 3), *Saccharomycopsis* showed a strong negative correlation with neryl acetate (ρ = −0.872, FDR = 0.0007) but positive correlations with pyridine (ρ = 0.688) and *γ*-butyrolactone (ρ = 0.602), indicating that this yeast primarily modulates aroma through ester hydrolysis rather than synthesis. Lactic acid bacteria (LAB) served as the principal ester biosynthesis drivers: *Enterococcus* correlated positively with sorbitan laurate (ρ = 0.886), γ-butyrolactone (ρ = 0.836), and dimethyl succinate (ρ = 0.707), while *Lactococcus* showed positive associations with 3-(methylthio) propionaldehyde (ρ = 0.871), 4-butyl-γ-butyrolactone (ρ = 0.818), and sorbitan laurate (ρ = 0.707).

**Table 3:** Top Spearman rank correlations between metagenomic genera and ester-related metabolites (|ρ| ≥ 0.7, FDR < 0.05)^*^.

Genus	Metabolite	ρ (rho)	*p*-value	FDR	Direction
*Enterococcus*	Sorbitan laurate	0.886	<0.001	0.0005	Positive
*Saccharomycopsis*	Neryl acetate	−0.872	<0.001	0.0007	Negative
*Lactococcus*	3-(Methylthio)propionaldehyde	0.871	<0.001	0.0007	Positive
*Pantoea*	Neryl acetate	0.843	<0.001	0.001	Positive
*Lactococcus*	1,3-Dicaffeoylquinic acid	−0.854	<0.001	0.001	Negative
*Enterococcus*	gamma-Butyrolactone	0.836	<0.001	0.002	Positive
*Lactococcus*	4-Butyl-gamma-butyrolactone	0.818	<0.001	0.002	Positive
*Streptococcus*	6”-O-Malonylglycitin	−0.814	<0.001	0.003	Negative
*Enterococcus*	4-Butyl-gamma-butyrolactone	0.807	<0.001	0.003	Positive
*Staphylococcus*	4-Pentenoic acid	−0.804	<0.001	0.003	Negative
*Mammaliicoccus*	6”-O-Malonylglycitin	−0.779	<0.001	0.006	Negative
*Streptococcus*	Neryl acetate	−0.771	<0.001	0.006	Negative
*Streptococcus*	1-Acetoxy-2-hydroxyheptadecenone	0.764	<0.001	0.007	Positive
*Mammaliicoccus*	2-Acetamido-2-deoxy-mannopyranose	0.757	0.001	0.008	Positive
*Pueraria*	Sorbitan laurate	−0.754	0.001	0.009	Negative
*Mammaliicoccus*	1-Acetoxy-2-hydroxyheptadecenone	0.750	0.001	0.009	Positive
*Enterococcus*	1-Acetoxy-2-hydroxyheptadecenone	0.736	0.002	0.012	Positive
*Weissella*	Neryl acetate	0.736	0.002	0.012	Positive
*Enterococcus*	6”-O-Malonylglycitin	−0.725	0.002	0.015	Negative
*Lactococcus*	Sorbitan laurate	0.707	0.003	0.019	Positive
*Enterococcus*	Dimethyl succinate	0.707	0.003	0.019	Positive
*Weissella*	Naringenin chalcone	0.707	0.003	0.019	Positive
*Pediococcus*	6”-O-Malonylglycitin	−0.707	0.003	0.019	Negative
*Pantoea*	6”-O-Malonylglycitin	0.704	0.003	0.020	Positive

^*^Per-sample genus abundance values (%) were derived from GTDB-Tk-classified metagenomic taxonomy. Per-sample metabolite intensities were extracted from untargeted LC–MS/MS metabolomics. Spearman rank correlation coefficients (ρ) and two-tailed p-values were computed across all 15 biological samples (3 replicates × 5 groups). Benjamini–Hochberg false discovery rate (FDR) correction was applied for multiple testing. Only correlations with |ρ| ≥ 0.7 and FDR < 0.05 are shown. “Positive” and “Negative” indicate the direction of correlation. Y, raw *P. thomsonii*; K, natural fermentation; S, yeast fermentation; B, bacterial fermentation; M, yeast-bacterial co-fermentation.

### 3.6 Analysis of aroma production

Table 4 presents the concentrations (μg/g) of volatile flavor compounds, including esters, alcohols, acids, aldehydes, ketones, phenols, and pyrazines, in Y and four fermented groups (K, B, S, M). Esters, crucial for fruity and wine-like aromas (64), increased significantly with fermentation, with co-fermentation exhibiting the highest levels. Ethyl linoleate in co-fermentation reached 1009.73 ± 32.51 μg/g, surpassing K (285.82 ± 8.59 μg/g), B (340.34 ± 10.76 μg/g), S (150.17 ± 5.82 μg/g), and was absent in Y. This high production likely stemmed from elevated lipase and esterase activities and a high free linoleic acid content in co-fermentation (9,12-octadecadienoic acid at 173.19 ± 5.27 μg/g) (65). Ethyl palmitate was also highest in co-fermentation (335.85 ± 9.76 μg/g), significantly more than in K (113.19 ± 2.97 μg/g), B (121.06 ± 3.10 μg/g), S (48.43 ± 1.59 μg/g), and Y (27.39 ± 0.65 μg/g), contributing sweet and waxy notes (66). Ethyl 9,12,15-octadecatrienoate reached 277.11 ± 7.95 μg/g in co-fermentation, much higher than in K (75.73 ± 2.59 μg/g) and S (48.09 ± 1.26 μg/g), while Y had only 15.21 ± 0.42 μg/g and B had none. Ethyl oleate appeared only in K (10.49 ± 0.31 μg/g) and co-fermentation (42.20 ± 1.19 μg/g), with co-fermentation being higher. (E)-9-Octadecenoic acid ethyl ester was present at 37.72 ± 1.01 μg/g in co-fermentation, followed by B (31.91 ± 0.99 μg/g), K (17.30 ± 0.52 μg/g), and S (7.11 ± 0.18 μg/g). Ethyl stearate reached 32.46 ± 1.02 μg/g in co-fermentation, while B was approximately 12.58 ± 0.37 μg/g and S was 6.30 ± 0.11 μg/g. Other esters, such as 3-hydroxybutanoic acid ethyl ester, appeared only in S and co-fermentation; isoamyl lactate appeared in B and co-fermentation; and 2-hydroxypropanoic acid ethyl ester was detected exclusively in co-fermentation (4.47 ± 0.03 μg/g). For alcohols, which offer wine-like and plant-like notes (67), benzyl alcohol was absent in Y but emerged post-fermentation, peaking in co-fermentation (39.09 ± 1.16 μg/g) and B (38.32 ± 0.16 μg/g), followed by S (23.91 ± 0.87 μg/g) and K (11.99 ± 0.37 μg/g). It likely originates from phenylalanine metabolism (68). Phenylethyl alcohol, via the Ehrlich pathway from phenylalanine (69), was highest in S (9.02 ± 0.22 μg/g), then B (8.28 ± 0.17 μg/g) and co-fermentation (7.40 ± 0.19 μg/g), with K lower (3.03 ± 0.01 μg/g). The slightly lower level in co-fermentation than S may indicate metabolic competition by bacteria. 2,3-Butanediol was not detected in Y but was abundant post-fermentation: K (88.31 ± 2.75 μg/g), co-fermentation (63.29 ± 1.95 μg/g), S (58.89 ± 1.72 μg/g), and B (38.19 ± 1.13 μg/g). The highest level in K may result from multiple 2,3-butanediol-producing bacteria in natural fermentation. 2-Furanmethanol, from sugar degradation (70), was highest in K (8.95 ± 0.20 μg/g), followed by co-fermentation (5.06 ± 0.11 μg/g), B (5.07 ± 0.13 μg/g), and S (2.31 ± 0.05 μg/g). Additionally, hexanal, with an odor threshold of approximately 5 μg/kg in aqueous matrices (71), was present at 3.61 ± 0.11 μg/g in Y, exceeding its sensory threshold and confirming its contribution to the undesirable beany odor. Its complete elimination in all fermented groups represents a sensory quality improvement. Overall, all fermentation groups produced abundant alcohols, with co-fermentation showing a balanced profile of benzyl alcohol, phenylethyl alcohol, and 2,3-butanediol, contributing floral, sweet, and creamy notes. Ethyl linoleate, while reaching >1000 μg/g in co-fermentation, has a relatively high odor threshold, and its aromatic contribution may be less pronounced than trace compounds such as phenylethyl alcohol (odor threshold ~7.5 μg/kg) (72).

**Table 4:** Profile of volatile flavor compounds in fermented *P. thomsonii* (μg/g)^*^.

Item	Compound	Retention time (min)	CAS	Content (μg/g)				
				**Y group**	**K group**	**B group**	**S group**	**M group**
Esters	Hexadecanoic acid, methyl ester	39.93	112–39-0	3.81 ± 0.03^a^	4.02 ± 0.09^a^	ND	ND	ND
	Hexadecanoic acid ethyl ester	40.77	628–97-7	27.39 ± 0.65^a^	113.19 ± 2.97^c^	121.06 ± 3.10^c^	48.43 ± 1.59^b^	335.85 ± 9.76^d^
	Octadecanoic acid, ethyl ester	45.21	111–61-5	3.46 ± 0.08^a^	12.67 ± 0.29^b^	12.58 ± 0.37^b^	6.30 ± 0.11^a^	32.46 ± 1.02^c^
	9,12,15-Octadecatrienoic acid ethyl ester	48.05	1191-41-9	15.21 ± 0.42^a^	75.73 ± 2.59^c^	ND	48.09 ± 1.26^b^	277.11 ± 7.95^d^
	Ethyl Oleate	45.66	111–62-6	ND	10.49 ± 0.31^a^	ND	ND	42.20 ± 1.19^b^
	(E)-9-Octadecenoic acid ethyl ester	45.83	6114-18-7	ND	17.30 ± 0.52^b^	31.91 ± 0.99^c^	7.11 ± 0.18^a^	37.72 ± 1.01^d^
	Linoleic acid ethyl ester	46.69	544–35-4	ND	285.82 ± 8.59^b^	340.34 ± 10.76^c^	150.17 ± 5.82^a^	1009.73 ± 32.51^d^
	Butanoic acid, 3-hydroxy-, ethyl ester	21.33	5405-41-4	ND	ND	ND	2.04 ± 0.02^a^	2.50 ± 0.03^b^
	Isoamyl lactate	23.47	19329–89-6	ND	ND	1.78 ± 0.02^a^	ND	1.79 ± 0.01^a^
	Propanoic acid, 2-hydroxy-, ethyl ester	15.67	97–64-3	ND	ND	ND	ND	4.47 ± 0.03
Alcohols	Benzyl alcohol	31.67	100–51-6	ND	11.99 ± 0.37^a^	38.32 ± 0.16^c^	23.91 ± 0.87^b^	39.09 ± 1.16^c^
	Phenylethyl Alcohol	32.60	60–12-8	ND	3.03 ± 0.01^a^	8.28 ± 0.17^b^	9.02 ± 0.22^b^	7.40 ± 0.19^b^
	2,3-Butanediol	22.96	513–85-9	ND	88.31 ± 2.75^d^	38.19 ± 1.13^a^	58.89 ± 1.72^b^	63.29 ± 1.95^c^
	2-Furanmethanol	25.58	98–00-0	ND	8.95 ± 0.20^d^	5.07 ± 0.13^b^	2.31 ± 0.05^a^	5.06 ± 0.11^c^
Acids	Hexanoic acid	30.81	142–62-1	21.01 ± 0.03^d^	23.88 ± 0.79^e^	15.35 ± 0.51^b^	ND	15.53 ± 0.57^c^
	n-Hexadecanoic acid	54.04	57–10-3	91.34 ± 2.86^a^	500.67 ± 16.23^d^	309.74 ± 9.57^c^	178.48 ± 5.62^b^	270.72 ± 8.26^c^
	Butanoic acid, 3-methyl-	25.94	503–74-2	ND	19.12 ± 0.03^a^	19.64 ± 0.05^b^	ND	ND
	Pentanoic acid, 3-methyl-	25.95	105–43-1	ND	ND	ND	3.82 ± 0.11^a^	15.51 ± 0.51^b^
	9,12-Octadecadienoic acid	46.91	60–33-3	ND	ND	37.25 ± 1.12^a^	201.74 ± 6.29^c^	173.19 ± 5.27^b^
	Succinamic acid	39.94	638–32-4	ND	ND	ND	ND	2.24 ± 0.03
Aldehydes	Hexanal	7.52	66–25-1	3.61 ± 0.11	ND	ND	ND	ND
	Butanal, 3-methyl-	39.39	590–86-3	6.15 ± 0.17^d^	2.58 ± 0.03^b^	ND	0.68 ± 0.00^a^	4.23 ± 0.10^c^
Ketones	Acetoin	13.83	513–86-0	38.58 ± 1.13^c^	ND	8.19 ± 0.11^b^	ND	ND
	2,3-Octanedione	25.71	585–25-1	ND	ND	ND	ND	1.35 ± 0.02
Phenols	Phenol, 4-ethyl-	39.06	123–07-9	ND	ND	ND	2.31 ± 0.03^a^	17.92 ± 0.57^b^
	4-Vinylphenol	43.91	2628-17-3	ND	ND	ND	ND	42.05 ± 1.33
Pyrazines	Pyrazine, trimethyl-	17.78	14667–55-1	ND	1.51 ± 0.02^a^	1.41 ± 0.02^b^	ND	ND
	Pyrazine, tetramethyl-	20.02	1124-11-4	ND	30.10 ± 0.92^c^	24.94 ± 0.72^b^	ND	17.58 ± 0.55^a^

*Values are presented as mean ± standard deviation (*n* = 3 biological replicates per group). Different superscript letters within each row indicate significant differences (*p* < 0.05). ND, not detected. Compounds were identified by matching mass spectra with the NIST 17 database (match factor > 800) and retention indices. Quantitation was performed using 2,6-dichlorotoluene as the internal standard, and concentrations are expressed as μg/g of dried sample. Retention time (min) and CAS registry numbers are provided for each compound. Y, raw *P. thomsonii*; K, natural fermentation; S, yeast fermentation; B, bacterial fermentation; M, yeast-bacterial co-fermentation.

Solid-state fermentation significantly changed the flavor compound profile in *P. thomsonii*. Co-fermentation transformed esters, phenols, alcohols, and acids, creating a complex flavor with fruity, floral, wine-like, and smoky notes. In contrast, natural fermentation produced higher levels of certain pyrazines and 2,3-butanediol but had a lower total ester content than co-fermentation. Single-strain fermentation with S or B resulted in less diverse flavor profiles. Therefore, under the tested conditions, co-fermentation effectively developed products with unique *P. thomsonii* flavor characteristics.

## 4 Conclusion

This study systematically compared the effects of different fermentation methods—raw *P. thomsonii* (Y), natural fermentation (K), yeast fermentation (S), bacterial fermentation (B), and yeast–bacterial co-fermentation (M)—on the nutritional, functional, and aromatic quality of *P. thomsonii* during solid-state fermentation (SSF). Using an integrated multi-omics approach comprising metagenomics, untargeted metabolomics, and volatilomics, we characterized the microbial community structure, functional gene profiles, metabolome, and volatile compound profile to achieve a systems-level understanding of the biotransformation mechanisms. The findings demonstrated that SSF significantly transformed the microecology and functional quality of *P. thomsonii*, with all fermentation methods reorganizing microbial communities and increasing CAZy gene abundance and enzyme activities to enhance polysaccharide degradation, proteolysis, and secondary metabolite formation.

Among the treatments, co-fermentation exhibited the highest *β*-glucosidase and cellulase activities, promoting efficient flavonoid aglycone release and cell wall polysaccharide degradation. Co-fermentation also offered superior nutritional and functional properties, with significantly higher levels of reducing sugars (15.45 ± 0.26 mg/g), total flavonoid content (TFC, 9.30 ± 0.17 mg RUT/g), total phenolic content (TPC, 13.19 ± 0.25 mg GAE/g), and crude protein (12.56 ± 0.26%) compared to other treatments. Additionally, co-fermentation improved the amino acid profile, achieving the highest total amino acids (TAA) with elevated levels of essential and umami-contributing amino acids. Moderate decreases in phenylalanine and tyrosine corresponded with increased flavonoid and phenolic acid accumulation. In terms of aroma, co-fermentation produced a desirable flavor compound profile, with significantly higher levels of esters, alcohols, and characteristic phenolics than other treatments. Although natural fermentation had the highest microbial diversity and produced certain pyrazines and 2,3-butanediol, it posed a potential risk of off-flavor or contaminant formation. Single-strain fermentations offered stable, controllable processes but were inferior to co-fermentation in integrated nutritional, functional, and flavor performance.

The multi-omics analysis provided mechanistic insights into these phenotypic outcomes. The co-fermentation reorganized the microbial community structure, with *Saccharomycopsis* and *Bacillus* effectively co-colonizing the substrate. Metagenomic sequencing identified 7,737 KO (KEGG Orthology) entries and 521 metabolic pathways, and revealed enrichment of CAZy families (GH1, GH13, CBM50) in co-fermentation, providing the genetic basis for the observed polysaccharide degradation and flavonoid deglycosylation. The enrichment of carbohydrate and amino acid metabolism pathways in co-fermentation was consistent with its superior nutritional profile. Untargeted metabolomics identified 1,693 metabolites, with co-fermentation uniquely enriching isoflavone aglycones, bioactive peptides, and esterase-related compounds. VIP analysis revealed that co-fermentation produced the greatest number of differentially expressed metabolites and the most pronounced upregulation, reflecting the strongest metabolic regulatory capacity among all treatments. The Spearman correlation analysis linked specific genera to aglycone production and ester formation, revealing that *Saccharomycopsis* primarily modulated aroma through ester hydrolysis, while LAB served as principal ester biosynthesis drivers.

From a food safety perspective, *S. fibuligera* and *B. velezensis* employed in the defined co-culture possess well-established safety credentials for food applications. In the present study, the co-fermentation system effectively suppressed opportunistic pathogens, specifically *Enterobacter* and *Klebsiella*, that dominated the natural fermentation microbiota, thereby enhancing the microbiological safety of the fermented product. Concurrent reductions in histidine and valine further indicated a diminished potential for biogenic amine formation, a critical safety consideration in fermented food systems. Additionally, hexanal, a volatile marker of lipid oxidation and a principal contributor to the undesirable beany odor, was completely eliminated across all fermented groups, representing a concurrent improvement in both sensory quality and chemical safety. Collectively, these safety-related outcomes, combined with the molecular identification of both strains via ITS and 16S rRNA gene sequencing, respectively, provide compelling evidence for the feasibility of utilizing these microorganisms in food production.

In summary, under the conditions of this study, co-fermentation achieved balanced nutritional and flavor-enhancing effects with favorable food safety attributes. These effects likely resulted from metabolic complementarity between yeast and bacteria and the co-enrichment of key enzyme systems. These findings provide a theoretical basis for developing high-value fermented foods from *P. thomsonii* and offer a reference framework for the precision microbial transformation of medicinal and edible homologous materials. Future research should focus on bioactivity evaluation and comprehensive, strain-specific safety assessment to support the industrial application of co-fermented *P. thomsonii* as high-value fermented foods.

## Data Availability

The 16S rRNA gene sequencing data with Bacillus velezensis NA03 have been deposited in the NCBI repository under accession number “PZ818998.1” (GenBank), the internal transcribed spacer (ITS) sequencing data with Saccharomycopsis fibuligera YPD01 have been deposited in the NCBI repository under accession number “PZ829667.1” (GenBank), and the metagenomic sequencing data have been deposited in the EBI Metagenomics repository under BioProject accession number “PRJEB123606” (https://www.ebi.ac.uk/ena/browser/view/PRJEB123606).

## Supplementary material

Supplementary Figure S1 Cell morphology and molecular identification of *Saccharomycopsis fibuligera* YPD01. **(a)** Cell morphology under light microscopy. **(b)** Internal transcribed spacer (ITS) region sequencing chromatogram and identification result. The strain was identified by ITS region sequencing, with sequence alignment against the NCBI nucleotide database confirming species identity as *S. fibuligera*.

Supplementary Figure S2 Cell morphology and molecular identification of *Bacillus velezensis* NA03. **(a)** Cell morphology under light microscopy. **(b)** 16S rRNA gene sequencing chromatogram and identification result. The strain was identified by 16S rRNA gene sequencing, with sequence alignment against the NCBI nucleotide database confirming species identity as *B. velezensis*.

Supplementary Figure S3 Standard curves for the quantitative determination of bioactive compounds and enzyme activities in fermented *P. thomsonii*. **(a)** Rutin standard curve for total flavonoid content (TFC) determination. **(b)** Gallic acid standard curve for total phenolic content (TPC) determination. **(c)** Maltose standard curve for α-amylase activity assay. **(d)** p-Nitrophenol standard curve for β-glucosidase activity assay. **(e)** Glucose standard curve for cellulase activity assay. Each curve was constructed using a minimum of six concentration gradient points, each measured in triplicate (*n* = 3 technical replicates per concentration). In all panels, the x-axis represents the standard concentration and the y-axis represents the absorbance measured at the specified wavelength. The linear regression equation, coefficient of determination (*R*²), and 95% confidence interval band are displayed in each panel.
