# Supplementary material for: Multi-omics insights into co-fermentation by Saccharomycopsis fibuligera and Bacillus velezensis enhancing the nutritional, metabolic, and aromatic quality of Pueraria thomsonii
Source: Front Nutr. 2026 Aug 27;13:1938235. doi: 10.3389/fnut.2026.1938235 (PMC13559536; doi:10.3389/fnut.2026.1938235)
Supplement: Supplementary file 1 [file Table_1.DOCX]

Supplementary Material

## 1.1 Supplementary Tables

**TABLE S1** Changes in biomass (lg copies/mg) of pure and co-culture systems of *S. fibuligera* YPD01 and *B. velezensis* NA03 with culture time

| Culture time（h） | Pure culture (lg copies/mg) | | Co-culture (lg copies/mg) | |
| --- | --- | --- | --- | --- |
|  | *S. fibuligera* YPD01 | *B. velezensis* NA03 | *S. fibuligera* YPD01 | *B. velezensis* NA03 |
| 0 | 5.99 | 7.17 | 5.87 | 7.25 |
| 12 | 6.63 | 8.51 | 7.38 | 9.05 |
| 24 | 7.43 | 8.96 | 7.83 | 9.06 |
| 36 | 7.43 | 8.16 | 8.39 | 9.79 |
| 48 | 7.67 | 8.05 | 8.16 | 9.29 |
| 60 | 7.78 | 7.94 | 7.88 | 9.19 |
| 84 | 7.55 | 7.74 | 7.69 | 8.96 |
| 108 | 7.02 | 7.18 | 7.56 | 8.77 |
